# Reduced Laughter Contagion in Boys at Risk for Psychopathy

**DOI:** 10.1016/j.cub.2017.08.062

**Published:** 2017-10-09

**Authors:** Elizabeth O’Nions, César F. Lima, Sophie K. Scott, Ruth Roberts, Eamon J. McCrory, Essi Viding

**Affiliations:** 1 Division of Psychology and Language Sciences, Department of Clinical, Educational and Health Psychology, University College London, Bedford Way, London WC1H 0AP, UK; 2 Faculty of Psychology and Educational Sciences, Parenting and Special Education Research Unit, KU Leuven, Leopold Vanderkelenstraat, Leuven, Belgium; 3 Division of Psychology and Language Sciences, Institute of Cognitive Neuroscience, Queen Square, University College London, London WC1N 3AR, UK; 4 Faculty of Psychology and Education Sciences, University of Porto, Rua Alfredo Allen, Porto, Portugal; 5 Instituto Universitário de Lisboa (ISCTE-IUL), Lisboa, Portugal

**Keywords:** disruptive behavior, callous-unemotional traits, psychopathy, social affiliation, social connectedness, positive vocalizations, laughter, emotional resonance, emotional contagion

## Abstract

Humans are intrinsically social animals, forming enduring affiliative bonds [1]. However, a striking minority with psychopathic traits, who present with violent and antisocial behaviors, tend to value other people only insofar as they contribute to their own advancement [2, 3]. Extant research has addressed the neurocognitive processes associated with aggression in such individuals, but we know remarkably little about processes underlying their atypical social affiliation. This is surprising, given the importance of affiliation and bonding in promoting social order and reducing aggression [4, 5]. Human laughter engages brain areas that facilitate social reciprocity and emotional resonance, consistent with its established role in promoting affiliation and social cohesion [6, 7, 8]. We show that, compared with typically developing boys, those at risk for antisocial behavior in general (irrespective of their risk of psychopathy) display reduced neural response to laughter in the supplementary motor area, a premotor region thought to facilitate motor readiness to join in during social behavior [9, 10, 11]. Those at highest risk for developing psychopathy additionally show reduced neural responses to laughter in the anterior insula. This region is implicated in auditory-motor processing and in linking action tendencies with emotional experience and subjective feelings [10, 12, 13]. Furthermore, this same group reports reduced desire to join in with the laughter of others—a behavioral profile in part accounted for by the attenuated anterior insula response. These findings suggest that atypical processing of laughter could represent a novel mechanism that impoverishes social relationships and increases risk for psychopathy and antisocial behavior.

## Highlights

• Psychopathic traits are associated with a lack of enduring affiliative bonds
• Listening to human laughter engages brain areas that facilitate emotional resonance
• Boys at risk of psychopathy have reduced neural/behavioral responses to laughter
• This could reflect a mechanism underpinning reduced social connectedness

## Teaser

Laughter promotes affiliation with others and engages brain areas that facilitate emotional resonance. O’Nions, Lima et al. report that boys at risk for psychopathy show decreased responsiveness to laughter across these brain areas. Research into positive emotions can contribute to our understanding of atypical social affiliation in psychopathy.

## Results and Discussion

Laughter is a universal expression of emotion [14, 15] used to maintain social bonds [6, 9]. It is a highly contagious behavior: it can be primed simply by listening to others’ laughter [16]. Such emotional contagion has been posited as a mechanism for facilitating the coupling of emotions and behavior within groups, increasing cooperation, cohesiveness, and social connectedness [6, 9, 10]. The social nature of laughter is evident in that an individual is up to 30 times more likely to laugh when with others than when alone [17]. Laughter also plays a role in the vicarious experience of positive emotions, and it triggers the endogenous opioid system, argued to be key for prosocial communication and social bonding in primates and other mammals [9, 18, 19]. Neuroimaging studies demonstrate that listening to laughter automatically recruits motor and premotor regions involved in the production of emotional expressions [6], including the precentral gyrus, supplementary motor area, inferior frontal gyrus, and anterior insula [7, 8, 10, 20]. This preparatory motor response is thought to facilitate joining in with others’ positive vocalizations during social behavior, representing a neural mechanism for experiencing these emotions vicariously and promoting social connectedness [9, 10]. These findings from typical individuals have established laughter as an ideal probe for examining atypical social affiliation and connectedness.

Individuals with psychopathy show a reduced capacity to develop social relationships founded on an enjoyment of prosocial interaction or concern for others’ well-being [3]. More broadly, individuals with persistent antisocial behavior show reduced prosocial functioning and act in way that violates the rights of other people [21]. Investigating potential mechanisms underpinning impoverished social connectedness in individuals at risk of psychopathy and persistent antisocial behavior has the potential to inform the design of therapeutic approaches to foster prosocial behavior in these individuals who incur substantial societal costs [22]. Remarkably, there has been no systematic neurocognitive investigation of potential mechanisms of impaired social connectedness in this group of people. Instead, research has focused on how individuals with psychopathic traits and persistent antisocial behavior process other people’s distress [23]. For example, extant research shows that adults with psychopathy and children at increased risk for psychopathy (those with disruptive behaviors and “callous-unemotional traits” [2]) show reduced neural and physiological responses to others’ fear and pain [23, 24, 25, 26]. However, unlike individuals with autism, they do not have difficulties taking the perspective of other people [27, 28]. Knowing what other people think but not resonating with their feelings facilitates the ability to manipulate and deceive others, in line with one’s own self-interest [29]. While prosocial emotions likely evolved to promote mutualistic social investment and collaboration within groups [30], their absence may represent an alternative adaptive strategy involving promotion of oneself at others’ expense [31, 32, 33]. Although previous research has addressed the underpinnings of increased behavioral aggression in those at risk for psychopathy and persistent antisocial behavior [34], it fails to fully account for the impoverished social affiliation also evident in this group of people [35].

We hypothesized that boys with disruptive behaviors would be less responsive to others’ laughter at the neural and behavioral levels, reflecting a potential mechanism underpinning impoverished social connectedness. Specifically, we hypothesized that compared to typically developing controls, boys with disruptive behaviors would show an attenuated subjective desire to join in with the laughter of others and reduced neural activation across premotor and motor areas involved in processing laughter and positive vocalizations: the precentral gyrus, supplementary motor area (SMA), inferior frontal gyrus (IFG), and anterior insula (AI) [7, 10, 11, 20]. These regions are implicated in auditory-motor integration and motor readiness to join in [7, 10, 11, 20]. We hypothesized that attenuated responsiveness to laughter across these regions would be particularly characteristic of boys with high levels of callous-unemotional traits and disruptive behaviors who show the most impoverished patterns of social affiliation. Finally, we hypothesized that neural responses to laughter across our regions of interest would in part explain differences in the subjective desire to join in with laughter. This could particularly be the case in the AI, given that, in addition to showing auditory-motor properties, the insular cortex is thought to play a role in linking action information with emotional or motivational experience [12, 13, 36] and in representing interoceptive information, providing the basis for subjective emotional awareness [13, 37].

Here we investigated behavioral and neural responses to laughter in 11- to 16-year-old boys with (1) disruptive behaviors and high callous-unemotional traits (N = 32); (2) disruptive behaviors and low callous-unemotional traits (N = 30); and (3) matched typically developing controls (N = 31). Groups were matched for IQ, age, handedness, ethnicity, and socioeconomic status (demographic information reported in STAR Methods). We recorded fMRI responses while participants listened to genuine laughter, interleaved with posed laughter and distractor crying sounds. Participants were instructed simply to attend to the stimuli to ensure that potential responses seen in premotor and motor systems could not be accounted for by task-related motor or decisional processes [7, 10, 20]. To assess whether group differences reflected reactivity to genuine laughter as a basic emotional cue, rather than higher-level processing of the social meaning of laughter, we also included posed laughter (which is more volitional, rather than spontaneous/involuntary [7]). After scanning, participants completed a behavioral task in which they evaluated each sound on two dimensions (presented in separate blocks) using a seven-point scale: (1) “How much does hearing the sound make you feel like joining in and/or feeling the emotion?” (a behavioral measure of subjective laughter contagion) and (2) “How much does the sound reflect a genuinely felt emotion?” (a behavioral measure of the ability to infer laughter authenticity). Measuring the discrimination between the two types of laughter at behavioral and neural levels allowed us to index the ability to infer the authenticity of the emotional state of the speaker (“emotional authenticity”) (see STAR Methods).

**Figure 1:**
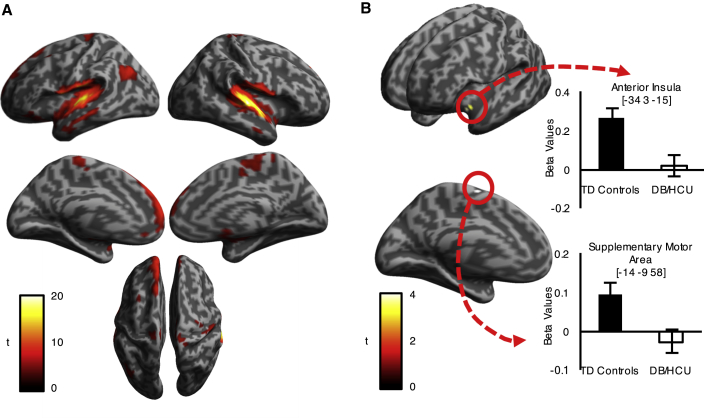
Neural Responses to Laughter across All Participants and Differences between Groups (A) Responses to genuine laughter versus rest across all participants, N = 93, p < 0.001 peak level uncorrected, family-wise error (FWE) corrected (p < 0.05) at cluster level. See also Table S1. (B) Responses to genuine laughter (versus rest) in typically developing (TD) boys versus boys with disruptive behavior and high callous-unemotional traits (DB/HCU) (thresholded at p < 0.05 small-volume corrected FWE). See also Table S3. Error bars represent the standard error of the mean.

Whole-brain analyses of responses to genuine laughter across all participants revealed activity across auditory, motor, and premotor, as well as limbic, medial pre-frontal and anterior temporal areas (Figure 1A; Table S1), consistent with previous studies [7, 10, 20]. When we compared responses for typically developing boys versus boys with disruptive behavior and high callous-unemotional traits, ROI analyses using small-volume family-wise error correction (SVC FWE) [38] revealed the predicted pattern of reduced response in boys with high callous-unemotional traits in the left AI (MNI coordinates for peak voxel: x = −34, y = 3, z = −15; *t*_(1,61)_ = 4.14, *z* = 3.87; p = 0.035, SVC FWE; cluster size = 46 voxels) (Figure 1B). In the SMA, differences were detected for typically developing boys versus disruptive boys with high callous-unemotional traits (MNI coordinates for peak voxel: x = −14, y = −9, z = 58; *t*_(1,61)_ = 4.14, *z* = 3.87; p = 0.043, SVC FWE; cluster size = 64 voxels; Figure 1B) and for typically developing boys versus disruptive boys with low callous-unemotional traits (cluster 1, MNI coordinates for peak voxel: x = 15, y = 6, z = 52; *t*_(1,59)_ = 4.42, *z* = 4.09; p = 0.02, SVC FWE; cluster size = 132 voxels; cluster 2, MNI coordinates for peak voxel: x = −14, y = −1, z = 52; *t*_(1,59)_ = 4.24, *z* = 3.95; p = 0.03, SVC FWE; cluster size = 101 voxels). For disruptive boys with low callous-unemotional traits, no group differences compared with typically developing boys were found in the remaining ROIs: the precentral gyrus, AI, and IFG. Follow-up analyses also indicated that the two groups of disruptive boys, those with high versus low callous-unemotional traits, did not significantly differ from each other in those ROIs that differentiated either group from the typically developing boys (all p > 0.16), and no additional group differences emerged in whole-brain comparisons.

**Figure 2:**
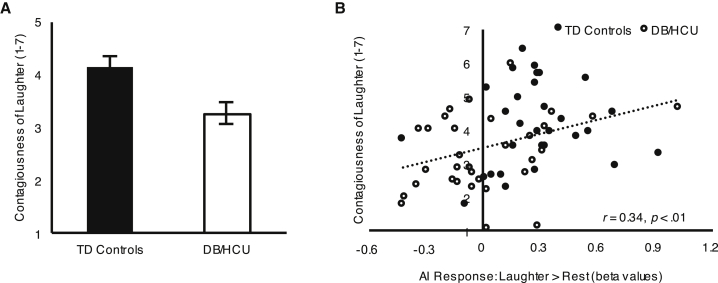
Group Differences on Perceived Contagiousness of Laughter and Relationship with Neural Responses in the Anterior Insula (A) Behavioral data on reported desire to join in with genuine laughter for TD versus DB/HCU boys (significant group difference: *t*(61) = 3.02, p < 0.01). Error bars represent the standard error of the mean. (B) Anterior insula response for genuine laughter versus rest (beta values extracted from a 10-mm sphere around the peak of the cluster) plotted against reported desire to join in with genuine laughter across TD and DB/HCU boys.

Behaviorally, boys with high callous-unemotional traits reported less desire to join in with genuine laughter compared to typically developing boys (Table 1; Figure 2A), whereas those with low callous-unemotional traits did not differ from typically developing boys or boys with high callous-unemotional traits (Table 1). Given the behavioral differences between typically developing boys and boys with high callous-unemotional traits, we also examined the relationship between their behavioral and brain data. We found a correlation between ratings of desire to join in with laughter and AI responses to laughter across the two groups (r = 0.34, p < 0.01; Figure 2B); in addition, importantly, AI responses to laughter mediated the effect of group on ratings of desire to join in with laughter. The total effect of group on ratings of desire to join in was −0.89 (95% confidence interval [CI]: −1.48, −0.30), and the indirect (mediated) effect through AI responses was −0.24 (95% CI: −0.57, −0.05), indicating that approximately 27% of the effect of group on desire to join in was mediated by AI responses [39] (full mediation model in Figure S1). No such mediation effect was detected in the SMA. Main analyses did not include covariates such as ADHD symptoms, on the basis that it is problematic to covary for variables intrinsically related to group assignment [40]. However, when analyses were re-run including ADHD symptoms as covariates, all group comparisons remained significant.

**Table 1:** Participant Characteristics and Questionnaire Data

	TD Controls	DB/HCU	DB/LCU	TD versus DB/HCU	TD versus DB/LCU	DB/HCU versus DB/LCU
	(N = 31)	(N = 32)	(N = 30)	p Value^a^	p Value^a^	p Value^a^
**Characteristics and Questionnaires**						

Age	13.92 (1.80)	14.66 (1.37)	14.42 (1.61)	p = 0.213^b^	p > 0.3	p > 0.3
Socio-economic status^c^	2.83 (1.12)	3.08 (0.82)	2.70 (1.17)	p > 0.3^b^	p > 0.3	p > 0.3^b^
F-IQ^d^	101.23 (12.37)	96.90 (11.36)	101.55 (14.18)	p > 0.3	p > 0.3	p > 0.3^b^
Verbal T score^d^	50.42 (8.54)	46.29 (9.31)	52.97 (11.19)	p = 0.221	p > 0.3^b^	p = 0.044
Performance T score^d^	50.61 (10.63)	49.71 (7.74)	48.24 (7.76)	p > 0.3	p > 0.3	p > 0.3
Ethnicity	18 white, 4 black, 9 mixed	17 white, 6 black, 9 mixed	20 white, 3 black, 7 mixed	p > 0.3	p > 0.3	p > 0.3
Handedness	26 right, 5 left	28 right, 4 left	29 right, 1 left	p > 0.3	p > 0.3	p > 0.3
Inventory of callous-unemotional traits^e^	24.81 (6.81)	51.19 (6.76)	32.75 (7.43)	p < 0.001	p < 0.001	p < 0.001
Conduct disorder symptoms^e^	0.68 (0.79)	11.44 (4.98)	5.43 (2.22)	p < 0.001^b^	p < 0.001^b^	p < 0.001^b^
ADHD symptoms^f^^,^^g^	12.60 (7.68)	25.60 (11.75)	22.94 (11.38)	p < 0.001^b^	p < 0.001^b^	p > 0.3
Generalized anxiety disorder symptoms^f^^,^^g^	3.66 (1.96)	9.25 (4.17)	8.43 (4.89)	p < 0.001^b^	p < 0.001^b^	p > 0.3
Major depressive symptoms^f^^,^^h^	3.19 (1.83)	6.89 (4.37)	5.79 (3.54)	p < 0.001^b^	p < 0.003^b^	p > 0.3
Alcohol use and disorders^i^	0.51 (1.47)	2.42 (3.92)	2.98 (5.46)	p = 0.041^b^	p = 0.068^b^	p > 0.3
Drug use and disorders^i^^,^^j^	0.13 (0.72)	2.13 (4.43)	3.34 (4.92)	p = 0.051^b^	p < 0.005^b^	p > 0.3
Self-rated pubertal development^i^^,^^k^	8.90 (2.86)	10.31 (2.87)	8.80 (3.87)	p = 0.171	p > 0.3^b^	p = 0.261

**Behavioral Responses to Laughter**						

Desire to join in with genuine laughter^l^	4.15 (1.20)	3.26 (1.14)	3.54 (1.20)	p = 0.011	p = 0.161	p > 0.3
Authenticity detection^l^	1.13 (0.83)	0.96 (0.76)	0.87 (0.79)	p > 0.3	p > 0.3	p > 0.3

Abbreviations: F-IQ, full IQ score calculated on two-subset Wechsler Abbreviated Scale of Intelligence; ADHD, attention-deficit/hyperactivity disorder; DB/HCU, boys with disruptive behavior and high callous-unemotional traits; DB/LCU, boys with disruptive behavior and low callous-unemotional traits.

a All p values are Bonferroni corrected and obtained from t tests, except for ethnicity and handedness (Bonferroni-corrected Fisher’s exact tests used).

b Welch’s t test used due to inhomogeneity of variance between groups.

c Missing data from three DB/LCU participants.

d Missing data from two participants (one DB/LCU and one DB/HCU).

e Measures taken at screening phase, comprising parent and teacher report.

f Measures taken at scanning session: parent report.

g Missing data from one DB/HCU participant.

h Missing data from two DB/LCU participants.

i Child self-report at scanning session.

j Missing data from one DB/LCU participant.

k Missing data from one TD and one DB/LCU participant.

l Assessed using a behavioral task at scanning session.

Finally, to examine whether reductions in brain responses related to affiliative rather than higher-level socio-cognitive processes, we contrasted cortical and behavioral discrimination between genuine and posed laughter. Across all participants, whole-brain analyses indicated that genuine laughter elicited stronger responses than posed laughter in the right temporal pole, right IFG, and left superior temporal gyrus (Table S2). These areas are consistent with previous studies on emotional authenticity processing in the auditory domain [7, 41]. They might be key for processing the prominent acoustic hallmarks that signal genuine laughter (e.g., higher pitch [6, 7, 42]) and for the higher-order socio-emotional and evaluative processes [43] needed to infer whether laughter is posed or genuine. Of the ROIs, increased responses to genuine laughter were only found in the IFG. No supra-threshold clusters were found for the contrast posed laughter > genuine laughter. At the set statistical thresholds, neural and behavioral discrimination between genuine and posed laughter was similar between typically developing boys versus boys with high callous-unemotional traits and between typically developing boys versus boys with low callous-unemotional traits (for behavioral discrimination, see Table 1; for neural discrimination, see Tables S3 and S4, which for completeness report results at p < 0.001 uncorrected, cluster size ≥ 10 voxels). Thus, the capacity to detect emotional authenticity at the neural and behavioral levels did not differ across the three groups.

As an additional control measure, we examined whether differences in basic auditory responses to laughter could account for the observed group differences in response to genuine laughter. There were no group differences in responses to laughter within primary auditory regions or within 10-mm spheres around auditory peaks revealed by the main effect of laughter across all participants (left: MNI coordinates: x = −46, y = −18, z = 1; right: MNI coordinates: x = 51, y = −10, z = −2), both for typically developing boys versus boys with high callous-unemotional traits and for typically developing boys versus boys with low callous-unemotional traits, suggesting no differences in how the groups responded to laughter at a basic auditory processing level.

These findings provide the first empirical evidence that boys with disruptive behavior show atypical neural responses to laughter, a primitive and potent social cue that plays a major role in facilitating social affiliation and promoting and maintaining social bonds. Boys with disruptive behavior and high callous-unemotional traits showed reduced responses in the AI, a region associated with automatic facilitation of motor responses to emotional vocalizations [10, 20], as well as with the experience of emotions and with linking action information with emotional and motivational processes [12, 13, 36]. Reduced AI responses to genuine laughter partially explained the lower subjective desire to join in with others’ laughter in boys with high callous-unemotional traits compared with typically developing boys. This suggests a link between AI response and the perceived contagiousness of laughter, which reflects its socio-emotional and motivational salience. More broadly, both groups of boys with disruptive behavior (irrespective of level of callous-unemotional traits) showed reduced responses in the SMA—also part of the network thought to facilitate the automatic priming of laughter when one hears other people laughing [6, 7, 11].

Our findings suggest that group differences in responses to genuine laughter were not attributable to difficulties in processing laughter at a basic auditory level or in discriminating different types of laughter (i.e., the capacity to infer social meaning). The latter finding is consistent with evidence of intact theory of mind ability in boys with disruptive behaviors [27], although it remains unclear which precise mechanism the boys with disruptive behaviors relied upon to infer authenticity: more basic detection of the acoustic markers that signal authenticity, higher-order socio-emotional and evaluative processes, or both combined. Additionally, the posed stimuli used here were generated by regular (untrained) speakers in a relatively artificial setting. These stimuli are typically perceived as natural and positive, but more research will be needed to determine whether similar findings would be obtained if we had used contextually appropriate posed laughter deployed by trained actors, for example.

Notably, in the present study, direct comparisons between disruptive boys with high and low callous-unemotional traits revealed no significant differences in neural response across ROIs that differentiated either group from typically developing boys. Although significantly reduced AI responses were only seen for the comparison between typically developing boys and boys with high callous-unemotional traits (and, as such, we ran mediation analysis on this group comparison only), we cannot firmly establish the selectivity of this finding to the high callous-unemotional group. It is, of course, possible that different developmental histories underlie atypical laughter processing in boys with high versus low callous-unemotional traits, something that warrants further investigation. Development of social connectedness is a bidirectional process, and the degree to which neural responses to laughter and subjective desire to join in with laughter are a consequence of atypical social connectedness versus experience-independent factors is unclear. This may also vary between children with high versus low callous-unemotional traits. Indeed, potential causes of reduced social connectedness that could give rise to atypical laughter processing might include the canalized development of an alternative social strategy centered on self-interested rather than collaborative behaviors, or various early life experiences or caregiver behaviors.

Limitations of the current study include the use of a research diagnosis of conduct disorder as a basis for identifying boys with disruptive behavior, as well as a focus on males. Replication of these findings in a clinically diagnosed sample is important, as well as investigation of potential gender differences. Additionally, our task did not allow us to investigate whether reductions in behavioral contagion and anterior insula response in boys at risk for psychopathy and persistent antisocial behavior were present for other positive emotional expressions. Future studies should address whether these findings are specific to laughter or extend to other types of positive vocalizations, for example sounds of achievement or pleasure [7, 20], or to non-vocal social gestures. Furthermore, future studies could include objective indices of contagion responses (e.g., facial electromyography), in addition to the self-report measure of motivation to join in with laughter that we used here. This could help elucidate whether the observed profile of behavioral responses reflects abnormalities in automatic motor contagion responses to laughter, in more subjective (conscious) components of emotional contagion, or both. The combined pattern of brain and behavioral results we obtained suggests that both might be involved. The areas where atypical responses were found, SMA and AI, are both part of the auditory-motor network that has been argued to support the automatic impulse to respond to the emotional expressions of others [7, 11, 20]. However, we could link perceived emotional contagion with activity in the AI only, not with SMA activity. Given that AI has been additionally implicated in emotional experience and subjective feelings [13, 37], this could mean that our behavioral measure is capturing conscious aspects of contagion better than more automatic motor resonance. Objective indices of motor resonance would potentially provide the additional sensitivity needed to detect whether the reduced SMA activity in boys with disruptive behaviors reflects atypical automatic motor contagion. Future studies could also include physiological responses such as heart rate and respiration to index arousal in response to laughter stimuli.

Despite these limitations, the current findings considerably extend our understanding of the neurocognitive processing of laughter in boys at risk for psychopathy. To date, explanations for the development of psychopathy have focused on the role of negative emotions, in particular deficits in processing other people’s distress [23]. Here we demonstrate that atypical processing of laughter, a potent positive social signal that plays a key role in social grooming and bonding [6, 9, 44], characterizes boys at risk for psychopathy and persistent antisocial behavior. This could represent a novel mechanism that may impoverish social relationships and potentiate a psychopathic trajectory, consistent with evolutionary accounts that suggest that psychopathy is an alternative strategy to mutualistic social investment driven by shared emotional experience and collaboration [32, 33, 34]. Alternatively, differences in neural responses to laughter could reflect a consequence of poor social connectedness over the course of development driven by aberrant caregiver signals. This may represent another possible risk pathway to persistent antisocial behavior. This study highlights the need for systematic longitudinal research to investigate the causal relationship between atypical responses to affiliative social cues and psychopathy. Such research would make it possible to explore the directionality of effects in different groups of children with disruptive behaviors and the degree to which these processes are under reciprocal influence. This, in turn, would motivate further inquiry into prevention and intervention components that may successfully promote the formation of affiliative bonds and reduce the risk of antisocial behavior.

## STAR★Methods

### Key Resources Table

**Table:** 

REAGENT or RESOURCE	SOURCE	IDENTIFIER
**Deposited Data**		

Data for each participant for group (typically developing, disruptive/high callous-unemotional traits, disruptive/low callous-unemotional traits), parameter estimates for bilateral anterior insula (AI) and supplementary motor area (SMA) regions of interest (ROIs) for the contrast genuine laughter versus baseline, and behavioral ratings of authenticity and contagion.	This paper	Data S1

**Software and Algorithms**		

Statistical Parametric Mapping (SPM, version 8)	[38]	http://www.fil.ion.ucl.ac.uk/spm/software/spm8/
WFU PickAtlas Toolbox with Automated Anatomical Labeling Atlas	[45, 46]	http://fmri.wfubmc.edu/software/PickAtlas; http://www.fil.ion.ucl.ac.uk/spm/ext/
Human Motor Area Template	[47]	http://lrnlab.org/
SPM anatomy toolbox	[48]	http://www.fil.ion.ucl.ac.uk/spm/ext/
MarsBaR	M. Brett et al., 2002, Conference on Functional Mapping of the Human Brain, abstract	http://marsbar.sourceforge.net/
Process	[49]	http://www.processmacro.org
Cogent 2000	Cogent 2000 team, Functional Imaging Lab/ Institute of Cognitive Neuroscience, UCL, UK	http://www.vislab.ucl.ac.uk/cogent_2000.php
Psychtoolbox	[50]	http://psychtoolbox.org/

### Contact for Resource Sharing

Further information and requests for resources should be directed to and will be fulfilled by the Lead Contact, Essi Viding (e.viding@ucl.ac.uk).

### Experimental Model and Subject Details

#### Participants

Boys aged 11-16 years were recruited from the community via newspaper advertisements, and local mainstream and specialist provision schools. Screening questionnaires were administered to parents of 360 boys and teachers of 215 boys whose families expressed an interest in taking part and provided informed consent. The screening measures yielded a research diagnosis of current conduct problems (our index of disruptive behavior); dimensional assessment of callous-unemotional traits; an overall psychopathology screen; demographic data for group-matching purposes (i.e., socioeconomic status, parent-defined ethnicity, and handedness); and information regarding previous neurologic or psychiatric diagnoses.

Current conduct disorder symptoms were assessed using the Child and Adolescent Symptom Inventory– 4R (CASI-4R) –Conduct Disorder (CASI-CD) subscale [51]. Callous-unemotional traits were assessed using the Inventory of Callous-Unemotional Traits (ICU) [52]. Both were scored by taking the highest ratings from either the parent or the teacher questionnaire for any given item [53]. For the CASI-CD scale, inclusion in the disruptive behavior group required that the score met either parent or teacher severity cut-off (parent report: cut-off = 4+ [ages 10–12] and 3+ [ages 12–16]; teacher report: cut-off = 3+ [ages 10–12], 4+ [ages 12–14], and 6+ [ages 15–16]). These scores are associated with a clinical diagnosis of conduct disorder [54]. Typically developing participants were required to score in the normal range for this measure, and below the atypical cut-off for total difficulties on the Strengths and Difficulties Questionnaire [55].

Automatic exclusion criteria for both disruptive and typically developing groups included a previous diagnosis of any neurological or psychotic disorder, or current psychiatric medication. To recruit a representative group of children with conduct problems, common comorbidities (ADHD, generalized anxiety disorder [GAD], depression, and substance/ alcohol abuse) were not used as exclusion criteria, but current parent-reported symptom counts were obtained during scanning sessions, so that their possible contribution to the findings could be systematically assessed.

On the basis of the screening information, one hundred participants took part in the fMRI scanning session. Participants were provided with a complete description of the study. Informed consent was obtained from parents and written assent from participants. All aspects of the study were approved by the University College London Research Ethics Committee (Project ID number: 0622/001) and work was conducted in accordance with the Declaration of Helsinki.

Two participants (one with disruptive behavior and one typically developing) withdrew from the session due to poor tolerance of the scanner environment. Data collection was terminated for a further two participants (both with disruptive behavior) due to fatigue. Of the sample who completed scanning (64 with disruptive behavior, 32 typically developing), data from three participants (two disruptive and one typical) was excluded due to poor compliance and lack of reliable auditory responses for task versus baseline. The remaining boys in the disruptive behavior group were designated into high and low callous-unemotional groups based on a median split of their scores on the ICU. All typically developing participants scored below the disruptive group median (42.24) on the ICU. Demographic and questionnaire data for participants are summarized in Table 1.

### Method Details

#### Psychometric and Questionnaire Measures

During the experimental session, participants completed the two-subtest version of the Wechsler Abbreviated Scale of Intelligence [56], and parents completed the full CASI-4R [51], the Alcohol Use Disorder Identification Test [57] and the Drug Use Disorder Identification Test [58]. Group differences were observed (Table 1) and were controlled for in subsidiary analyses.

#### Experimental Stimuli

The laughter stimuli consisted of 30 genuine and 30 posed laughs. We used vocalizations produced by several male and female speakers previously validated and used in behavioral and neuroimaging experiments [7, 59]. They were generated by six speakers (three female) in a sound-proof anechoic chamber at University College London. Genuine laughter was elicited using an amusement induction situation in a social interactive setting: speakers were shown video clips, which they had identified beforehand as amusing and that would easily cause them to laugh aloud; the experimenters, who had known all the speakers for a long time, interacted with them throughout the recording session to promote the naturalness and the social nature of the laughs (as they occur between friends in everyday interactions). Procedural details are provided elsewhere [7, 59]. For posed laughter, the speakers were asked to simulate laughter in the absence of any external stimulation, and they were encouraged to make it sound natural and positive. Genuine and posed laughs were matched for duration (genuine laughs, M = 2,461 ms; posed laughs, M = 2,296), and pilot data (N = 12) confirmed that genuine laughs are perceived as highly authentic (M = 5.94, on an authenticity scale from 1 to 7; posed laughs, M = 3.27). Thirty crying sounds were also included in the experiment as an emotional distractor condition, so that participants were less likely to detect that the manipulation concerned laughter specifically. Crying sounds consisted of a mix of genuine and posed stimuli and were produced by the same speakers as the laughs.

#### fMRI Acquisition and Procedure

A Siemens Avanto 1.5-T MRI scanner (Siemens Medical, Erlangen, Germany) using a 32-channel birdcage head coil was used to acquire a 5.5 min three-dimensional T1-weighted structural scan, and multislice T2^∗^-weighted echo planar volumes with blood-oxygen level-dependent contrast. The echo planar imaging sequence was designed to optimize signal detection and reduce dropout in the orbitofrontal cortex and amygdala [60]. Acquisition parameters were as follows: 42 2-mm slices acquired in an ascending trajectory with a 1-mm gap (voxel size = 3 × 3 x 2 mm); TE = 50 ms; slice repetition time = 87 ms, TR = 3654ms; slice tilt = 25°+/−5° (T.C); flip angle = 90°; field of view = 192 mm; phase oversampling = 12%.

Participants were told that they would hear different kinds of sounds, and that they should listen carefully to them. They were reminded that they should keep their head and face as still as possible throughout the experiment, and their eyes should be open. They were reminded that they did not need to press any response buttons or make decisions about the sounds (passive listening paradigm). Throughout the experiment, participants were monitored via an in bore camera to ensure that they were alert and staying still.

The sounds were presented in one run of 230 echo-planar whole-brain volumes lasting 14 min. The first 5 volumes of the run were discarded to allow longitudinal magnetization to reach equilibrium. Auditory onsets occurred after a 1.5 s (±0.5 s jitter) fixation period and, on each trial, participants listened to 3 randomly selected sounds of the same type. There were 75 trials in total: 30 of genuine laughter, 30 of posed laughter, 10 of crying sounds, plus 5 rest/silence trials. The sounds were presented in a pseudo-randomized order for each participant, and we ensured that no more than 3 trials of the same type were consecutively presented. Each of the 60 laughter sounds was presented three times during the experiment, and each of the 30 crying sounds was presented once. Sounds were played using Psychtoolbox [50] via a Sony STR-DH510 digital AV control center (Sony, Basingstoke, UK) and MRI-compatible insert earphones (Sensimetrics Corporation, Malden, MA, USA). Noise attenuation was achieved through careful fitting and insertion of correctly sized silicone headphone tips, and custom made foam ear cushions adjusted to accommodate the participant’s head.

#### Post-Scanning Behavioral Task

After the scanning, participants made behavioral ratings for each of the sound stimuli presented during the fMRI task. For one task, participants were asked to rate “contagion”: whether listening to the sound made them feel like joining in and/or feeling the emotion. For the second, they were asked to rate “authenticity”: whether they thought that the sounds were real or posed/faked. For the authenticity task, participants were informed that half of the sounds were in fact real and half were posed/faked. Participants made ratings for each stimulus using a seven-point scale. Sounds were played using Cogent 2000 (Cogent 2000, Functional Imaging Lab/ Institute of Cognitive Neuroscience, UCL, UK) via a Dell Latitude 3330 laptop (Dell, Dublin, Ireland) using AERO 7 Active Noise Cancelling Headphones (7dayshop, Guernsey).

For both tasks, stimuli were presented in a random order across six blocks, each consisting of fifteen stimuli. At the end of each set, participants could take a break before proceeding to the next. Each sound stimulus was presented for its duration, after which a question mark appeared on the screen, and participants could make their response. The response scale was visible throughout the stimulus presentation and response period (3000 ms). After participants made their response, the selected option was indicated on the screen for 750 ms. Subsequently, a fixation cross was presented for 500 ms before the presentation of the next stimulus. Task order (i.e., contagion versus authenticity) was pseudo-randomized across participants, and matched across groups. Authenticity was calculated as an index of discrimination by measuring the effect size (Cohen’s *d*) of the authenticity rating difference for genuine laughter and posed laughter stimuli within each participant. Contagion reflected the mean absolute rating for each participant. Internal consistency for the genuine laughter contagion measure was α = 0.93. For the authenticity measure, internal consistency was α = 0.87 for genuine laughter and α = 0.84 for posed laughter. Behaviorally, all groups rated genuine laughs as significantly more authentic than posed ones (*p*s < 0.001) and, crucially, there were no group differences in the magnitude of such discrimination (Table 1).

### Quantification and Statistical Analysis

#### fMRI Analysis

Scanning data were analyzed using Statistical Parametric Mapping software (SPM version 8; Wellcome Trust Centre for Neuroimaging, UK). Functional images were realigned to the first image, co-registered to the structural image, and spatially normalized to MNI space using parameters acquired from segmentation [61]. They were then resampled to 2 × 2 x 2 mm voxels and smoothed with an 8 mm Gaussian kernel. In order to check our data for motion artifacts, we used a custom script detecting between-volume movements greater than 0.5mm or 1 degree of rotation. Where movements were detected, the scan in which the movement occurred and the seven scans surrounding it were manually inspected for visible motion artifacts. In addition, first level masks were visually inspected for motion-related distortions. Volumes showing visible motion-related distortions were removed and interpolated using adjacent scans to prevent distortions of the between-subjects mask. Interpolated scans were then regressed out in the first-level design matrix. Visible motion-related distortions were found for 16 participants (typically developing, n = 4; disruptive/high callous-unemotional, n = 9; disruptive/low callous-unemotional, n = 3), and always constituted less than 10% of each participant’s data.

Event-related responses were modeled using the canonical hemodynamic response function, with event onsets modeled from the acoustic onset of the first stimulus in each trial to the offset of the third stimulus. Each condition was modeled as a separate regressor in a generalized linear model at the first level (single-subject), and six movement parameters (3 translations, 3 rotations) were also included as regressors of no interest. An additional regressor was included for participants with interpolated scans. The rest/silence trials and the fixation periods were used as implicit baseline. For each participant, T-contrast images were created for the following comparisons: (1) genuine laughter > baseline, (2) genuine laughter > posed laughter, and (3) posed laughter > genuine laughter. These images were then entered into second-level models: 1-sample t tests were used to examine effects across all participants; and 2-sample t tests were used to compare typically developing with disruptive/high callous-unemotional boys, and typically developing with disruptive/low callous-unemotional boys. Whole-brain main effects across all participants are reported for the contrasts Genuine Laughter > Rest (Table S1) and Genuine Laughter > Posed Laughter (Table S2). Whole-brain condition x group interactions for the contrast Genuine Laughter > Rest and Genuine Laughter > Posed Laughter are presented comparing typically developing controls to disruptive boys with high callous-unemotional traits (Table S3) and comparing typically developing controls to disruptive boys with low callous-unemotional traits (Table S4).

#### Regions of Interest (ROIs)

For comparisons between groups, we conducted ROI analyses within regions for which we had a priori hypotheses, based on previous fMRI experiments of nonverbal emotional vocalizations [7, 10, 20]. For the precentral gyrus and inferior frontal gyrus, we used the standard (bilateral) anatomical masks from the Automated Anatomical Labeling (AAL) atlas in the WFU PickAtlas Toolbox for SPM [45, 46]. The same atlas was used for the insula, but we modified the original anatomical mask to include all voxels y > 0, on the basis of evidence that responses to nonverbal vocalizations peak in the anterior portion of this region [7, 10, 20]. The supplementary motor area (SMA) ROI included pre-SMA and SMA-proper, and it was defined using the Human Motor Area Template, which was created by combining results of a meta-analysis of 126 functional studies with anatomical guidelines [47]. For the control analysis within primary auditory regions, we used the SPM Anatomy Toolbox to delineate regions TE1.0, TE1.1 and TE1.2 of bilateral auditory cortex [48].

#### Brain-Behavior Associations

For the analysis of brain-behavior associations, we used the MarsBaR Toolbox (M. Brett et al., 2002, Conference on Functional Mapping of the Human Brain, abstract) to extract data within small spheres (10mm radius) centered on the peaks of the effect of group. The mediation analyses were computed using Process [49]; we estimated total, direct, and indirect effects of group on perceived laughter contagiousness (including neural responses as mediators), and inference was based on bootstrap bias corrected 95% confidence intervals (95% CIs were estimated using a bias corrected bootstrap method, 20,000 samples). The full mediation model is shown in Figure S1.

### Data and Software Availability

Data for each participant for group (typically developing, disruptive/high callous-unemotional traits, disruptive/low callous-unemotional traits), parameter estimates for bilateral anterior insula (AI) and supplementary motor area (SMA) regions of interest (ROIs) for the contrast genuine laughter versus baseline, and behavioral ratings of authenticity and contagion are provided as an excel file (Data S1). Due to ethical restrictions, we are unable to provide demographic or questionnaire data for individual participants from which they could potentially be identified or identify themselves.

## Author Contributions

E.O., C.F.L., E.V., and E.J.M. developed the study concept and design, and S.K.S. provided critical comments. E.O. and R.R. collected the data. C.F.L. and E.O. performed the data analysis and interpretation under the supervision of E.V., E.J.M., and S.K.S. E.O., C.F.L., E.V., and E.J.M. drafted the manuscript, and E.V., E.J.M., E.O., C.F.L., and S.K.S. provided critical revisions. All authors approved the final version of the manuscript for submission.

## Supplemental Information

Document S1. Figures S1 and Tables S1–S4

Data S1. Summary Data for Each Participant (Information on Group Membership, Parameter Estimates for Regions of Interest, and Behavioral Ratings), Related to STAR Methods Data are provided for each participant for (1) group membership (i.e., typically developing, disruptive/high callous-unemotional traits, disruptive/low callous-unemotional traits), (2) parameter estimates for bilateral anterior insula (AI) and supplementary motor area (SMA) regions of interest (ROIs) for the contrast genuine laughter versus baseline, and (3) behavioral ratings of authenticity and contagion.

Document S2. Article plus Supplemental Information
