# Supplementary material for: Reduced Laughter Contagion in Boys at Risk for Psychopathy
Source: Curr Biol. 2017 Oct 9;27(19):3049–3055.e4. doi: 10.1016/j.cub.2017.08.062 (PMC5640510; doi:10.1016/j.cub.2017.08.062)
Supplement: Document S1. Figures S1 and Tables S1–S4 [file mmc1.pdf]

Current Biology, Volume *27*

Supplemental Information

Reduced Laughter Contagion

in Boys at Risk for Psychopathy

Elizabeth O'Nions, César F. Lima, Sophie K. Scott, Ruth Roberts, Eamon J. McCrory, and Essi Viding

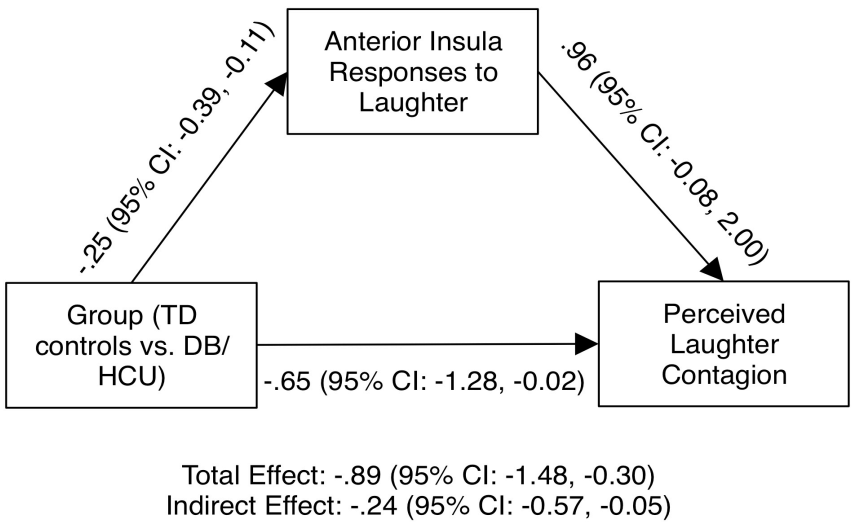

Figure S1. Model illustrating how anterior insula responses to laughter significantly mediated the effect of group (TD controls vs. DB/HCU) on perceived laughter contagion, Related to STAR Methods.
Inference was based on bootstrap bias corrected 95% confidence intervals (95% CIs were estimated using a bias corrected bootstrap method, 20,000 samples).

Region	MNI Peak Coordinates x	y	z	# voxels	*t*	*z*	FWE-corrected *p* value
R Superior Temporal Gyrus	51	-10	-2	12,170	20.27	> 8.00	< .001
R Superior Temporal Gyrus	62	-15	4		19.39	> 8.00	
R Middle Temporal Gyrus	60	-4	-27		4.76	4.49	
L Superior Temporal Gyrus	-46	-18	1	13,870	18.33	> 8.00	< .001
L Superior Temporal Gyrus	-64	-10	3		12.04	> 8.00	
L Middle Temporal Gyrus	-64	-10	-15		6.92	6.19	
L Inferior Frontal Gyrus	-42	26	-18		6.32	5.74	
L Insula	-39	0	-17		6.12	5.60	
L Temporal Pole	-39	8	-21		5.78	5.33	
L Middle Temporal Gyrus	-54	3	-29		5.04	4.72	
L Temporal Pole	-40	17	-24		4.77	4.50	
L Temporal Pole	-27	8	-26		4.43	4.20	
L Rolandic Operculum	-38	-7	15		4.00	3.83	
L Superior Medial Gyrus	-8	57	21	6,192	6.75	6.07	< .001
L Superior Medial Gyrus	-6	62	13		6.66	6.00	
L Superior Medial Gyrus	-4	51	37		6.45	5.84	
L Superior Medial Gyrus	-12	54	28		6.28	5.71	
L Superior Medial Gyrus	-14	32	54		6.12	5.59	
R Superior Medial Gyrus	8	57	24		4.67	4.41	
L Mid Orbital Gyrus	-4	56	-8		4.65	4.40	
R Superior Medial Gyrus	8	62	12		4.33	4.12	
L Rectal Gyrus	-8	39	-15		4.27	4.07	
L Middle Frontal Gyrus	-33	17	55		3.57	3.45	
L Mid Orbital Gyrus	0	48	-11		3.57	3.45	
L Paracentral Lobule	-10	-24	66	2,111	5.08	4.76	< .001
R Supplementary Motor Area	10	-25	55		4.59	4.35	
R Supplementary Motor Area	8	-15	55		4.39	4.17	
R Postcentral Gyrus	12	-34	66		4.38	4.16	
R Paracentral Lobule	6	-27	63		4.24	4.04	
R Middle Cingulate Cortex	10	-10	48		3.90	3.74	
L Paracentral Lobule	0	-21	58		3.32	3.22	
R Hippocampus	22	-9	-17	1,464	5.73	5.29	< .001
R Parahippocampal Gyrus	21	8	-24		3.69	3.55	
L Angular Gyrus	-45	-72	33	1,416	5.70	5.26	.029
L Angular Gyrus	-39	-60	25		4.63	4.38	
L Amygdala	-18	-6	-15	582	4.50	4.27	.017
L Hippocampus	-21	-16	-17		4.15	3.96	
L Hippocampus	-26	-19	-11		3.68	3.54	

Table S1. Whole-brain main effect across all participants (N = 93) for the contrast Genuine Laughter > Rest, Related to Figure 1A and STAR Methods. Results are thresholded at a voxel-wise threshold of $p < .001$ (uncorrected), family-wise error (FWE) corrected for multiple comparisons at the cluster level ($p < .05$). We report a maximum of 15 gray matter local maxima (that are more than 8mm apart) per cluster

Region	MNI Peak Coordinates x	y	z	# voxels	*t*	*z*	FWE-corrected *p* value
R Temporal Pole	52	3	-6	1,318	7.64	6.70	.001
R Inferior Frontal Gyrus	48	24	-8		3.85	3.70	
R Inferior Frontal Gyrus	54	30	1		3.55	3.42	
L Superior Temporal Gyrus	-50	-7	-6	611	5.08	4.76	.006
L Superior Temporal Gyrus	-42	-18	-5		3.92	3.76	

Table S2. Whole-brain main effect across all participants (N = 93) for the contrast Genuine Laughter > Posed Laughter, Related to STAR Methods. Results are thresholded at a voxel-wise threshold of $p < .001$ (uncorrected), family-wise error (FWE) corrected for multiple comparisons at the cluster level ($p < .05$). We report a maximum of 15 gray matter local maxima (that are more than 8mm apart) per cluster. No supra-threshold clusters were found for the reverse contrast, Posed Laughter > Genuine Laughter.

Contrast	Region	MNI Peak Coordinates			# voxels	*t*	*z*	*p* value (peak uncorrected)
		x	y	z				
Genuine Laughter > Rest								
TD > DB/HCU	R Superior Parietal Lobule	16	-46	54	208	4.52	4.18	< .001
	L Supplementary Motor Area	-14	-9	58	69	4.14	3.87	< .001
	R Inferior Parietal Cortex	44	-39	16	265	4.07	3.81	< .001
	R Superior Temporal Gyrus	54	-39	15		3.45	3.28	.001
	L Superior Temporal Gyrus	-52	5	-14	126	3.91	3.68	< .001
	L Inferior Frontal Gyrus	-51	33	-8	105	3.90	3.67	< .001
	R Middle Temporal Gyrus	39	-57	12	83	3.88	3.65	< .001
	R Precentral Gyrus	38	-9	57	111	3.86	3.64	< .001
	L Inferior Temporal Gyrus	-40	-22	-21	40	3.82	3.60	< .001
	L Superior Temporal Gyrus	-66	-34	19	24	3.81	3.60	< .001
	L Inferior Frontal Gyrus/Insula	-21	17	-15	81	3.71	3.51	< .001
	L Inferior Frontal Gyrus	-34	24	-17		3.61	3.42	< .001
	L Middle Occipital Gyrus	-44	-70	6	118	3.66	3.46	< .001
	L Hippocampus	-27	-12	-11	24	3.65	3.46	< .001
	R Fusiform Gyrus	46	-34	-17	20	3.64	3.45	< .001
	L Hippocampus	-28	-13	-21	14	3.64	3.45	< .001
	R Postcentral Gyrus	34	-39	55	36	3.58	3.40	< .001
	R Inferior Frontal Gyrus	50	20	18	23	3.57	3.39	< .001
	R Supplementary Motor Area	10	-10	57	33	3.55	3.37	< .001
	R Supplementary Motor Area	15	-1	52	15	3.53	3.36	< .001
	L Postcentral Gyrus	-38	-39	54	15	3.52	3.34	< .001
	R Supplementary Motor Area	10	-25	58	39	3.47	3.30	< .001
	R Supplementary Motor Area	2	-22	60		3.33	3.18	.001
	L Supplementary Motor Area	-4	0	54	22	3.46	3.29	.001
	R Middle Occipital Gyrus	40	-85	18	11	3.40	3.24	.001
	R Postcentral Gyrus	34	-28	55	19	3.38	3.23	.001
	R Superior Occipital Gyrus	27	-67	33	13	3.34	3.19	.001
Genuine Laughter > Posed Laughter								
TD > DB/HCU	L Superior Parietal Lobule	-28	-60	62	381	4.90	4.48	< .001
	L Cerebellum	-18	-54	-29	157	4.26	3.97	< .001
	R Cuneus	21	-81	45	198	4.25	3.96	< .001
	R Superior Occipital Gyrus	27	-75	34		3.55	3.37	< .001
	R Superior Parietal Lobule	24	-63	60	198	4.01	3.77	< .001
	R Superior Parietal Lobule	32	-58	60		3.73	3.53	< .001
	R Postcentral Gyrus	36	-39	63	38	3.85	3.63	< .001
	L Inferior Frontal Gyrus	-40	17	28	29	3.77	3.56	< .001
	R Insula Lobe	39	2	9	36	3.75	3.54	< .001
	L Superior Frontal Gyrus	-21	-6	58	53	3.66	3.46	< .001
	L SupraMarginal Gyrus	-64	-42	30	28	3.63	3.44	< .001
	R Superior Frontal Gyrus	22	-1	57	57	3.61	3.42	< .001
	R Superior Frontal Gyrus	33	-4	61		3.25	3.11	.001
	L Inferior Temporal Gyrus	-57	-22	-23	14	3.51	3.34	< .001
	R Inferior Frontal Gyrus	44	9	9	10	3.47	3.30	< .001
	L Middle Frontal Gyrus	-45	39	22	11	3.40	3.24	.001
	R Superior Occipital Gyrus	27	-88	27	15	3.37	3.22	.001

Table S3. Whole-brain Condition x Group interactions for the contrasts Genuine Laughter > Rest and Genuine Laughter > Posed Laughter, comparing typically developing controls (TD, N = 31) and disruptive boys with high callous-unemotional traits (DB/HCU, N = 32), Related to Figure 1B and STAR Methods. Results are thresholded at a voxel-wise threshold of $p < .001$ (uncorrected), cluster size ≥ 10 voxels. We report a maximum of 15 gray matter local maxima (that are more than 8mm apart) per cluster. No supra-threshold clusters were found for the comparison DB/HCU > TD controls across the two contrasts.

Contrast	Region	MNI Peak Coordinates x	y	z	# voxels	*t*	*z*	*p* value (peak uncorrected)
Genuine Laughter > Rest								
TD > DB/LCU	R Supplementary Motor Area	15	6	52	229	4.42	4.09	< .001
	R Superior Frontal Gyrus	16	12	46		4.07	3.80	< .001
	L Supplementary Motor Area	-14	-1	52	101	4.24	3.95	< .001
	L Inferior Frontal Gyrus	-56	21	4	122	4.03	3.77	< .001
	R Middle Temporal Gyrus	50	-42	-3	20	3.79	3.57	< .001
	L Precentral Gyrus	-52	-1	43	78	3.79	3.57	< .001
	L Inferior Frontal Gyrus	-30	33	3	41	3.68	3.48	< .001
	L Superior Orbital Gyrus	-16	53	-9	28	3.67	3.47	< .001
	R Inferior Frontal Gyrus	42	36	3	14	3.49	3.31	< .001
Genuine Laughter > Posed Laughter								
TD > DB/LCU	R Middle Frontal Gyrus	36	6	37	399	4.45	4.12	< .001
	R Inferior Frontal Gyrus	51	8	27		4.34	4.03	< .001
	R Precentral Gyrus	42	2	31		3.38	3.22	.001
	R Superior Frontal Gyrus	18	8	54	220	4.42	4.09	< .001
	R Middle Frontal Gyrus	32	54	4	232	4.15	3.87	< .001
	R Middle Frontal Gyrus	39	59	0		3.28	3.13	.001
	R Middle Frontal Gyrus	33	29	31	140	4.12	3.85	< .001
	R Middle Frontal Gyrus	39	39	30		3.51	3.33	< .001
	L Inferior Parietal Cortex	-68	-25	25	55	4.02	3.77	< .001
	R Inferior Frontal Gyrus	40	33	-2	39	3.72	3.51	< .001
	R Anterior Cingulate Cortex	3	20	28	141	3.69	3.49	< .001
	R Anterior Cingulate Cortex	9	27	28		3.63	3.44	< .001
	L Anterior Cingulate Cortex	-3	26	24		3.44	3.27	.001
	L Middle Frontal Gyrus	-32	47	9	23	3.50	3.33	< .001
	R Inferior Frontal Gyrus	44	8	6	12	3.49	3.32	< .001
	R Superior Parietal Lobule	28	-63	60	23	3.46	3.28	.001
	R Middle Temporal Gyrus	56	-48	4	10	3.41	3.25	.001
	L Caudate Nucleus	-6	11	9	13	3.41	3.24	.001
	L Superior Parietal Lobule	-24	-69	54	13	3.37	3.21	.001
DB/LCU > TD	R Cerebellum	18	-63	-38	13	3.60	3.41	< .001
	L Precentral Gyrus	-24	-21	76	11	3.51	3.33	< .001
	R Amygdala	22	-15	-5	11	3.48	3.30	< .001

Table S4. Whole-brain Condition x Group interactions for the contrasts Genuine Laughter > Rest and Genuine Laughter > Posed Laughter, comparing typically developing controls (TD, N = 31) and disruptive boys with low callous-unemotional traits (DB/LCU, N = 30), Related to STAR Methods.

Results are thresholded at a voxel-wise threshold of $p < .001$ (uncorrected), cluster size ≥ 10 voxels. We report a maximum of 15 gray matter local maxima (that are more than 8mm apart) per cluster. No supra-threshold clusters were found for the comparison DB/LCU > TD for the contrast Genuine Laughter > Rest.
